# Performance and Navigation Behavior of using Teleportation in VR First-Person Shooter Games

**DOI:** 10.1145/3661133

**Published:** 2024-08-30

**Authors:** ANIRUDDHA PRITHUL, HUDSON LYNAM, EELKE FOLMER

**Affiliations:** Computer Science and Engineering, University of Nevada, Reno, Reno, United States; Computer Science and Engineering, University of Nevada, Reno, Reno, United States; Computer Science and Engineering, University of Nevada, Reno, Reno, United States

**Keywords:** Virtual reality, locomotion, teleportation, performance, human navigation behavior

## Abstract

Teleportation has been adopted as the preferred mode of navigation in many virtual reality (VR) experiences due to its ability to allow users to easily move beyond the limitations of the tracking space while minimizing the risk of inducing VR sickness. Teleportation instantly translates the user’s viewpoint to a user-selected destination and therefore eliminates any optical flow generation that could cause visual-vestibular conflict. Though teleportation is a discrete navigation method unique to the domain of VR, most VR experiences are modeled after three-dimensional experiences found on desktop/console platforms that use continuous locomotion. How the use of teleportation affects the performance and navigation behavior of its users, especially in competitive first-person shooter environments is unknown, yet it could have a significant effect on gameplay design. We conducted a user study (*n* = 21) that compares teleportation versus continuous locomotion using a VR first-person shooter game with other players being simulated using AI agents. We found significant differences in performance, navigation behavior, and how both locomotion methods are perceived by its users. Specifically, using teleportation, players traveled farther but during combat were found to be more stationary and as a result got hit more frequently. These differences were profound and carry the potential to impact multiplayer games. We discuss possible strategies to balance gameplay.

## 1 INTRODUCTION

For navigation in **virtual reality (VR)**, walking using positional tracking arguably offers the most natural immersive experience with the lowest chance of inducing VR sickness as it fully resembles navigation in the real world. Unfortunately, available walking space is typically constrained by various factors. VR systems relying on outside-in tracking typically only support limited tracking spaces (i.e., 4.5 × 4.5 m). Available tracking space for recent consumer VR headsets (i.e., Oculus Quest 2) that rely on visible light inside-out tracking, is defined by the available space inside consumers’ homes, which is generally limited. Thus users may only free up enough space to meet the minimum tracking requirements. With the virtual environment often larger than the available tracking space, an ongoing research challenge for the past decades has been to develop **alternative locomotion techniques (ALT)** [3] that allows users to travel beyond tracking space confines in a pragmatic way. Of the many suggested solutions, teleportation, and continuous locomotion both have gained large-scale adoption and can be most commonly found in most VR experiences.

Using continuous locomotion, users regulate their movement velocity using their controller, such as a thumbstick or touchpad. Simultaneously, the direction of their movement is dictated by the orientation of their headset, ensuring that they move in the direction they are looking. This control scheme mirrors the three-dimensional (3D) navigation mechanics commonly found in desktop and console gaming platforms. The player’s heading is typically managed using a mouse or a thumbstick, while directional movement is controlled through a keyboard or the other thumbstick on a game controller.

Though continuous locomotion is most familiar to VR users, a major limitation of this method is that it often induces VR sickness. When navigating, optical flow is generated [15], but the absence of any vestibular and proprioceptive afferents that are normally generated when walking can lead to sensory conflict [9]. VR sickness is a type of motion sickness unique to VR and may include symptoms such as nausea, pallor, sweating, stomach awareness, increased heart rate, drowsiness, disorientation, and general discomfort [26]. Teleportation is considered a far safer locomotion method, as it discontinuously changes a user’s viewpoint and thus avoids any optical flow generation and visual-vestibular conflict. Several studies have found the use of teleportation to significantly reduce VR sickness incidence [11, 21, 24]. Offering teleportation as a locomotion method can be seen as an accessibility feature that could broaden the number of individuals who can partake in the VR experience. VR experiences will likely continue to offer multiple locomotion methods to cater to different user preferences and needs.

Consumer VR devices are currently primarily marketed toward gamers. Multiplayer **first-person shooters (FPS)** are one of the most popular and commercially successful game genres on PC/console platforms with popular franchises like the Call of Duty series having sold more than 400 million copies.^1^ Bringing FPS to VR comes with significant challenges due to a fundamental difference in how players could be navigating. Differences in movement time, accuracy, effort, and presence have been suggested to exist between teleportation and continuous locomotion [36]. For multiplayer experiences, there is the issue that a user’s avatar is discontinuously represented when using teleportation (i.e., users jumping), which can be confusing for bystanders [20]. Discontinuous travel could alter intended gameplay,^2^ but in multiplayer FPS games, it is very important to be able to predict other players’ trajectories to improve shooting accuracy and avoid getting hit by enemy fire. Also, teleportation over longer distances is significantly faster than continuous locomotion. However, teleportation requires users to first select a destination, whereas, with continuous locomotion, movement is fluid and more easily controlled. In FPS games, fast movement is a defining feature.

Continuous locomotion offers users unrestricted 360° freedom of movement. However, the navigation behavior of users who employ teleportation has not been thoroughly examined or analyzed. It remains uncertain whether users would exhibit similar behaviors when utilizing continuous locomotion. The ability to move laterally relative to the player’s forward direction, known as strafing, is a vital skill in FPS games. It enables players to maintain a continuous line of fire on their opponents while simultaneously avoiding their attacks, thereby enhancing performance and accuracy. Nonetheless, it is currently uncertain whether users would engage in strafing movements while employing teleportation, similarly to how they would with continuous locomotion. In the context of teleportation, strafing behavior without continuous forward motion would need to involves lateral virtual movements, such as sidestepping to evade attacks. While players have the capability to physically move sideways, it is uncertain whether the use this option. If they do not, then it could significantly impact their performance in the virtual environment. Positional tracking is now widely available on consumer VR platforms, and both locomotion techniques are to be used in conjunction with physical movement, but a recent study found that tracking space utilization using teleportation is extremely limited [35], with users mostly standing still. As VR shifts from single to multiplayer experiences, such as FPS games, it becomes increasingly important to comprehend how the limitations of teleportation impact navigation behavior and gameplay. Few studies have focused on analyzing and understanding navigation behavior between both locomotion techniques.

This article’s user study comparing teleportation and continuous locomotion in an FPS game yields valuable insights. It examines performance, the relationship between physical and virtual movement, navigation behavior (including strafing behavior), and tracking space utilization, which is useful to understand how large a tracking space should be. Additionally, it evaluates how users perceive both techniques, when using it and when other players use it. These findings significantly enhance our understanding of using teleportation and continuous locomotion in FPS games, which finds various implications for gameplay design.

## 2 RELATED WORK

Virtual locomotion has been an active area of research. Al-Zayer et al. provide [3] a comprehensive overview of various locomotion techniques including walking-in-place, treadmills, redirected walking, and so on. First mentioned in an academic paper by Mine et al. [29], teleportation has been around for more than two decades. Teleportation is often used to benchmark against other ALTs [12, 13, 24]. Various ways to improve teleportation have also been proposed [5, 8, 14, 22, 23, 28, 39]. A comprehensive review of teleportation improvements and comparative studies can be found here [34]. We limit our discussion to work most closely related to this article. Several approaches blend the mechanics of discontinuous teleportation with continuous locomotion. Dash [6] is a teleportation method that aims to reduce spatial disorientation by providing a small amount of optical flow during the viewpoint transition to allow for path integration. Compared to regular teleportation, Dash enables path integration and reduces spatial disorientation without increasing VR sickness. Griffin et al. [23] proposes a new version of teleportation that alternates between first- and third-person perspectives. This assures a continuous avatar representation but without generating any optical flow that could cause VR sickness. Translation snapping [16] moves the user in small increments or snaps from one position to another, with controls similar to that of continuous locomotion. Because this is considered a series of small teleports, it does not generate optical flow and avoids VR sickness. Similarly, HyperJump [1] blends the advantages of teleportation with continuous locomotion. This technique splits continuous locomotion into successive jumps at half-second intervals. HyperJump enables quicker navigation while maintaining spatial awareness and orientation.

A criticism of many locomotion studies with teleportation is that they often only involve simple navigation or search tasks [33] with observed performance and usability not necessarily being representative of their real-world usage. Consumer VR systems come with two controllers, and VR experiences like games require players to use both controllers simultaneously to perform various time-critical tasks like shooting at enemies or interacting with objects. It has been argued that overloading the hands with navigation functionality could significantly impede performance and increase cognitive load [27]. Griffin et al. [24] evaluated performance, presence, usability, cognitive load, and VR sickness incidence of controller-based locomotion methods like teleportation and continuous locomotion to controller-less techniques like walking-in-place [38] and head tilt [37]. This study was done under realistic real-time conditions typically found in games, where players had to perform a bimanual shooting task while simultaneously navigating to collect ammo. Results surprisingly showed no difference between the four ALTs in terms of enemies killed and shooting accuracy, which suggest that there might not be a penalty associated with navigating and shooting using the same controller as was the case with the hands-busy techniques. Players traveled the largest distance using continuous and the lowest using teleportation, yet teleportation allowed for picking up the largest amount of ammo. Players using teleportation remained largely stationary and took more damage than when using continuous. Teleportation had the lowest presence and VR sickness incidence. Monteiro et al. [30] evaluates continuous locomotion using an FPS with four different types of enemies but no comparison to other ALTs is made.

Hashemian et al. [25] compares three ALTs (continuous with a controller, head-tilt, stepping/leaning) to real walking with users being seated for head-tilt and continuous. During a navigation task, participants were required to collect balloons using their controllers. Real walking provided the best performance and user experience, while leaning interfaces outperformed continuous with a controller. This study suggests using a controller for both interaction and navigation impedes performance, and this contradicts earlier findings by Griffin et al. [24], though firing a gun is very different from grabbing a balloon, which requires more physical interaction. This study finds performance is impeded when a controller is used for both interaction and navigation, challenging the previous results of Griffin et al. [24], who found no difference. However, using a controller to grab balloons demands significantly more physical effort compared to using it to fire a gun, which could explain this difference.

The bulk of current VR experiences are still single-user, but with the increasing popularity of titles like VR Chat, Rec Room, and Gorilla Tag, there is a trend toward multi-user VR experiences. There is limited research on the application of teleportation within environments involving multiple users. Group navigation techniques offer ways to navigate the virtual environment together, either while the users are co-located or connected over the network. Weissker et al. [40] presented a comprehensive overview of various group navigation techniques, such as steering [4] and multi-ray jumping [41], which lets multiple users teleport together in an orderly manner.

What locomotion method is used can have a profound effect on resulting user experience [7], but this has predominantly been evaluated from the perspective of the user and not from that of any bystanders. Few studies have evaluated locomotion in a multi-user setting. Most closely related to our article is the work by Freiwald et al. [19]. In this study, authors compare teleportation to continuous locomotion using a two-player snowball game with a specific focus on evaluating how each method affects co-presence and subjective fairness. A user study (*n* = 18) had participants play the game in pairs against each other with one participant using teleportation and the other continuous locomotion. Players were required to pick up a snowball and throw it at the other player. Results found continuous locomotion to provide a significantly higher sense of co-presence and subjective fairness. Users were hit more using continuous locomotion with no difference in distance traveled between both methods. In follow-up research [20], authors propose four different avatar transition techniques and user study with AI-controlled avatars compare these to regular teleport and found these to increase spatial awareness.

Moore and Lagos [31] present two new ALTs that have been specifically designed for fast paced games. **Repeated short range teleport (RSRT)** appears to be an implementation of translation snapping [16] allowing users to repeatedly teleport a fixed distance, while **continuous movement pads (CMP)** lets users define a number of waypoints and then travel through these sequentially. A user study evaluates both ALTs to regular teleportation and continuous locomotion using an FPS like game with participants navigating through a series of levels while encountering AI enemies. Results are a mixed bag with RSRT and CMP doing worse in terms of level completion than continuous locomotion and teleportation, but CMP was most preferred by its users and had the highest navigation accuracy while RSRT scored the worst on these criteria. This research is most closely related to our article yet differs significantly in its approach, utilizing fixed path navigation and concentrating on evaluating two new ALTs. Our article offers a in-depth examination of differences between the two most widely adopted ALTs: teleportation and continuous locomotion specifically within the context of free-from navigation.

## 3 USER STUDY

Given the increasing interest in multi-user VR experiences, we are interested in comparing teleportation to continuous locomotion to understand whether any differences exist. Prior studies [19, 24] already elicited some differences but did not specifically focus on multiplayer FPS games, though this genre is highly popular on PC and console platforms and is becoming increasingly popular on VR platforms (i.e., Half-Life Alyx, Pistol Whip, Onward, and so on, are among the most popular games on VR platforms, with Half-Life Alyx selling more than 2 million copies.^3^ A key finding from a preliminary study [24], which we believe warrants further investigation, is that users utilizing teleportation tend to remain more stationary. It would be good to further substantiate these differences in comparison to continuous locomotion, for example, how much longer users stand still and how this affects total physical and virtual movement. For FPS games, we hypothesize that these differences in navigation behavior could impact their performance in two significant ways: First, it could increase their vulnerability to enemy attacks (i.e., hits taken), and, second, it could enhance their precision in shooting. Given that most VR games, including multiplayer FPS, typically provide both teleportation and continuous locomotion as navigation options, it becomes essential to understand how these methods are perceived not only by the players using them but also by others in the game. For instance, with teleportation, users may appear to be ‘jumping’ around the game environment, a movement that differs significantly from continuous locomotion. Should there be significant differences in navigation behavior, performance, and perception, this could significantly affect gameplay, and adjustments may be required to maintain balance and fairness. Ensuring an optimal gaming experience hinges on identifying and understanding such disparities. We formulated the following hypotheses:

- **H**_**1**_ : Differences in navigation behavior exist between teleportation and continuous locomotion.
- **H**_**2**_ : Differences in performance exist between teleportation and continuous locomotion.
- **H**_**3**_ : Teleportation and continuous locomotion are perceived differently (by its users and others).

### 3.1 Virtual Environment and Task

**Fig. 1.**
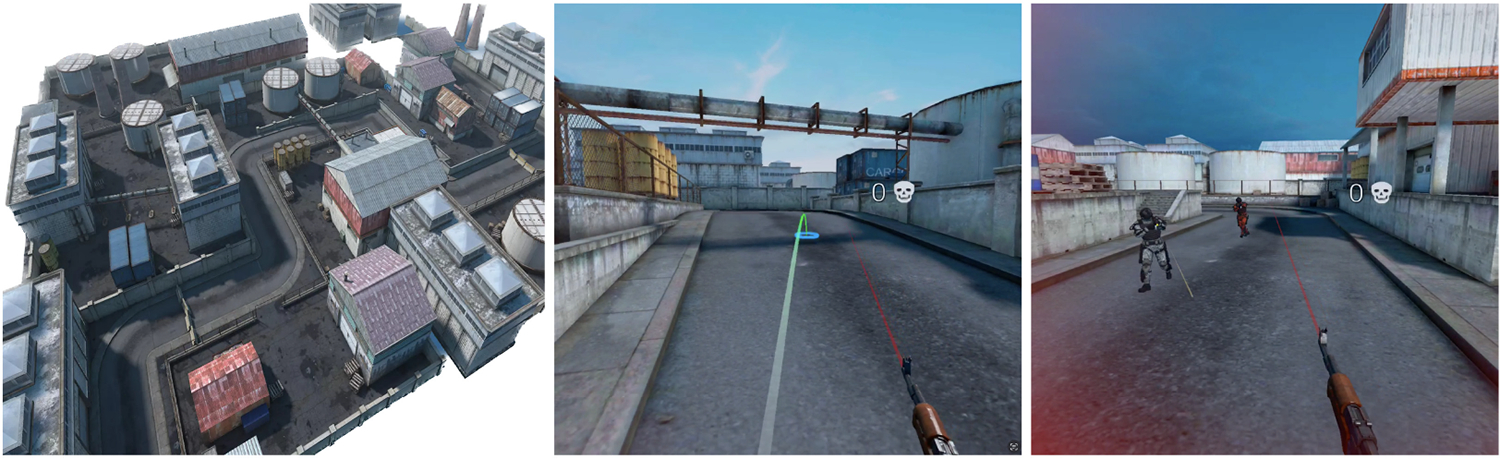
Left: Birdseye view of the virtual environment used in our experiment featuring an industrial look with open areas. Center: Teleportation arch shown as well as kill counter. Right: Visual feedback when a player is getting hit by bullets.

We designed our VE and task to resemble playing a popular multiplayer FPS like Call of Duty. We used an existing game asset^4^ that consisted of open areas featuring an industrial look with varying elevations (see Figure 1 (left)). There were walls and various objects to hide behind. Several invisible spawn points for the AI agents were placed in the periphery of the environment. When an AI agent is killed a new one spawns at one of these spawn points in a round-robin manner. The participants were tasked with killing as many AI agents as possible within a fixed time limit. Our game did not let participants die. This was to prevent participants who were not good at playing FPS games from getting frustrated by having to start over and over and to allow for a more controlled experience. Participants were not informed that they could not die but we encouraged them to kill as many enemies as possible while avoiding getting shot. To notify the participant of being shot at, a red vignette texture was shown in their peripheral vision as depicted in Figure 1 (right). This visual cue acted as a reinforcement mechanism to enhance their performance. Conversely, when the participants shot one of the agents, the agent’s material would flash a white color and it would emit particles with numbers telling the amount of damage dealt. Other particle effects like muzzle flashes and bullet trails as well as audio effects for gunshots and footsteps were implemented to make the experience more engaging. We also added a kill counter as a **head-up display (HUD)** element that increased every time the user killed an enemy. HUDs are considered to break presence in VR, but we believe its usage here is justified to encourage players to play the game better. Moreover, we were not measuring the presence felt by the users while playing.

### 3.2 Design of AI Agents

For our study, we needed to use a multi-user VR environment, but there are significant limitations to using real humans in terms of ensuring conditions are the same across participants. Apart from difficulties regarding organizing experiment sessions with multiple users at the same time in a shared VE and developing a capable multiplayer game, there is also the problem of ensuring a fixed difficulty level. Results from a real multiplayer game can be hard to interpret as each user will face opponents of varying skill levels and thus find the game to be more or less difficult. Thus, similar to closely related work [20], we opt for using AI bots instead, which can model consistent human behavior at scale. Our goal is to compare the use of both locomotion methods used by other agents (who can be either AI or other human users) from the user’s perspective. Since we lack understanding of how humans use teleportation in a FPS game (in fact finding that out was one of the key goals of this experiment), we have developed the AI to behave identically for both methods. This approach enables us to isolate and analyze the differences resulting from the distinct locomotion methods being utilized. The AI behaves in a humanlike way to make their behavior intuitive and highly realistic to the user.

**Fig. 2.**
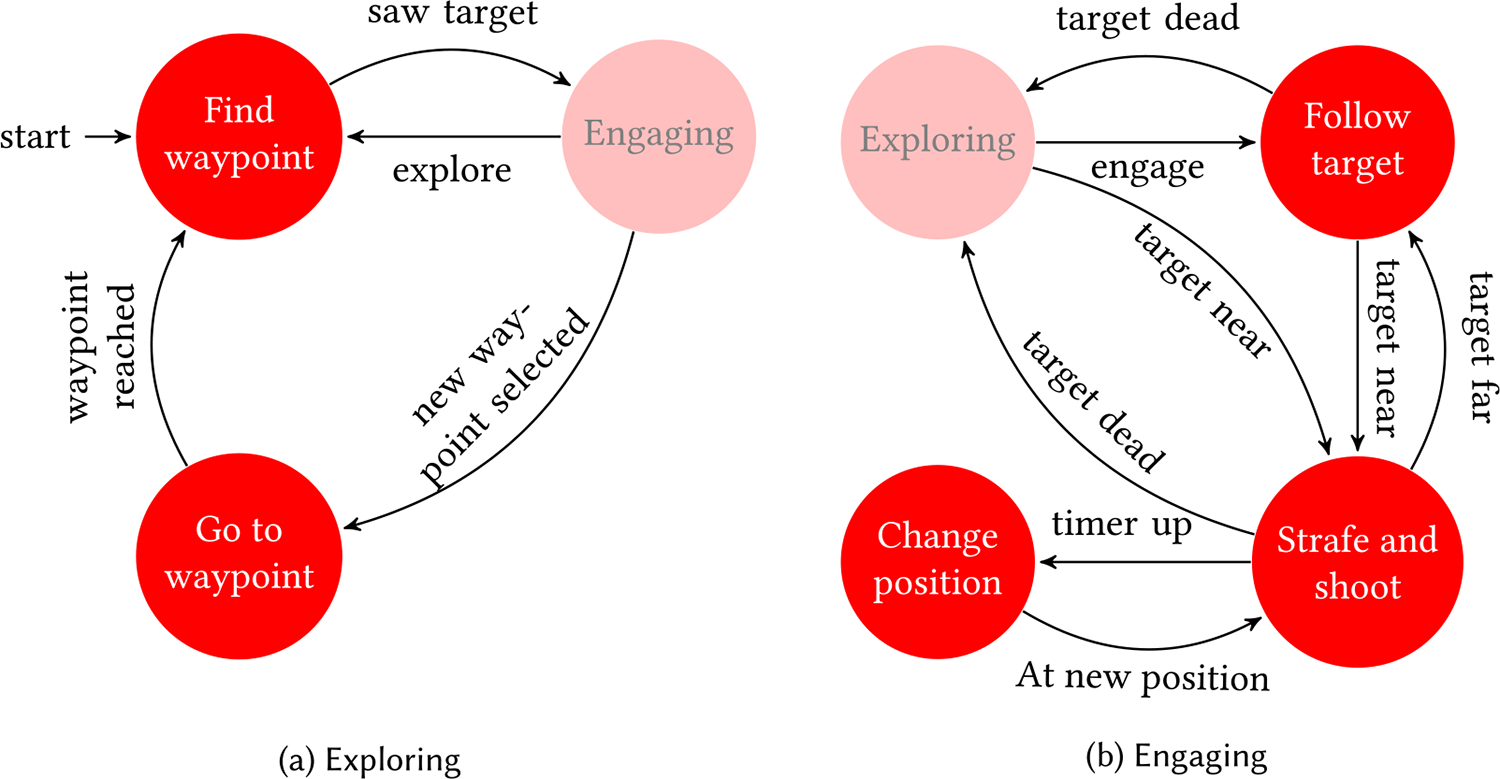
Finite state machine depicting a high-level behavior of the AI agent. Panel (a) shows the sub-states of the exploring state. The AI moves from one node to another random node that is connected to it. The agent moves to the engaging state once it sees a target (another agent or the player). Panel (b) shows the sub-states of the engaging state. The agent follows the target to get near, and once it gets close enough, it starts shooting and strafing around it at the same time. A random timer decides when the agent re-positions itself. It continues this behavior until the target is dead, at which point it goes back to the exploring state.

Figure 2 shows a state machine of the behavior pattern of the AI. Initially, upon spawning, the AI is in explore mode. It randomly selects a target on the map and travels toward it. While traveling, it may see another human or AI agent and start following it. When in range, the agent will start shooting. Upon being hit, the AI bot will engage in attacking its attacker and be in an engaged state. Strafing is a commonly observed practice in multiplayer FPS. Because strafing is an important behavior to simulate, we decided to let the AI randomly strafe short distances and change its position around the target intermittently. The teleportation distance for AI agents was set to range from 4 to 10 m. And in continuous mode, AI agents moved at a speed of 3.5 m, the same as the human user.

We used Unity’s inverse kinematics system to make the AI agent gradually point its gun at the desired target. This was done to make it possible to evade AI agents’ shots by moving around. A ray cast from the tip of the gun in its forward direction would determine if a target could be hit. If the ray cast reached its target, then the agent would start firing (i.e., hitscan). In terms of visual design, we used a rigged soldier asset from the Unity Asset Store. A bright orange material was used to make the agents visible from far away. The agents played appropriate animations while running and shooting. After extensive play-testing of the AI, we believe they resembled realistic human behavior in an FPS.

### 3.3 Instrumentation

The experiment was conducted using the Meta Quest 2 headset, which is a self-contained VR headset that does not need to be tethered to an external computer. It relies on camera-based inside-out tracking so no external trackers are needed either. It has a display with a per-eye resolution of 1832 × 1920 that runs at either 60, 72, 90, or 120 Hz and has a horizontal **field-of-view (FOV)** of 104°. We used the experimental 120 Hz mode in our study. We developed the VR environment in the Unity3D game engine. According to a survey^5^, the average size of tracking space available for VR users is 2.5 × 2.5 m, so we utilized this size for our tracking space.

### 3.4 User Locomotion

We used the Unity XR Toolkit plugin,^6^ which comes with default implementations of teleportation and continuous locomotion and that we modified as follows:

- *Teleportation*. We used the projectile mode for the teleportation target selection arch. The projectile’s speed was adjusted to 13 m/s to have a max teleport distance of around 18 m (as selecting destinations further away is very difficult anyway). Teleportation was activated using the trigger of the controller. Teleportation would be performed once the user lets go of the trigger. A custom blue circular reticle was used to convey the destination of the teleportation action.
- *Continuous locomotion*: We modified this implementation as follows. The script is designed to process input from two controller axes, each with a value range of [−1, 1]. It computes a 2D vector from these inputs, which is then clamped to a maximum magnitude of 1.0. The magnitude of this vector [0,1] is then linearly interpolated to correspond to a velocity value within the range of [0, 3.5] limiting the maximum movement speed to 3.5 m/s, which was determined experimentally. The velocity is set to the HMD’s local coordinate space after it is projected to the ground plane. Because continuous locomotion can induce VR sickness, we used an off-the-shelf plugin^7^ to implement a FOV restrictor. FOV restrictors reduce peripheral optical flow perception and minimize VR sickness incidence [17]. A recent study did not find the usage of a FOV restrictor to affect spatial navigation performance [2], though it may impede ability to detect enemies. The thumb stick was used to move in the left, right, forward, and backward directions. The input was applied in the user’s local coordinate space, with the head gaze direction specifying the forward direction.

All variables were determined experimentally using play testing and in such a way that it made the locomotion techniques most intuitive to use.

### 3.5 Participants

We recruited 21 participants for our study. These included 2 females, 1 non-binary, and 18 males with an average age of 25.14, SD = 3.8, and were all undergraduate or graduate students. Two subjects self-reported non-correctable impairments in perception or limitations in mobility but these were identified to be minor and not to affect their ability to participate in the study. We encouraged people that considered themselves to be avid gamers to participate in our study though this was not a specific inclusion criterion. Table 1 lists participant’s experience with VR, the usage of teleportation, multiplayer games, and multiplayer VR games on a scale of five levels that ranged from “no experience” to “lots of experience.” The study was IRB-approved and participants received a $10 gift card.

### 3.6 Procedure

We used a within-subjects design with locomotion mode as the independent variable. The two locomotion modes were continuous locomotion and teleportation. To control for order effects, we counterbalanced the order of independent variables tested using a Latin square. At the start of the experiment, participants verbally agreed to participate through a consent form. After this, they were provided with a unique ID, and a short description was given about the VR headset and the task they would be performing in the experiment. Participants were informed that if they felt VR sick during the experiment, then they could withdraw from the experiment at any time. Next, the participant put on the VR headset. We made sure it fit comfortably, and we allowed participants to adjust their interpupillary distance. After that, the tutorial environment was launched. Once placed in this scene, they had a gun in their right hand by default. Locomotion was assigned to the left hand and was set to teleport by default. A settings screen allowed them to switch hands and select a different locomotion method. Four blue dummy targets were placed around them. Shooting the targets would make them flash red. We encouraged participants to try out both locomotion methods and practice shooting at the dummies.

When participants felt comfortable with both locomotion methods, they could select their ID and shoot the start button upon which they were moved to our EV. Participants with odd IDs started with teleportation and participants with even IDs started with continuous locomotion. Participants were instructed to kill as many agents and take as little damage as possible. After 5 minutes, the first session of the experiment ended. The locomotion mode automatically toggled, and after a 5-s delay, a new session was started. Like before, this session lasted for another 5 min. Once the second session ended, participants were instructed to take off the headset. They then filled in a questionnaire intended to collect subjective feedback.

### 3.7 Data Collection

During the two sessions, we collected the following data.

#### 3.7.1 Quantitative Data:.

- Number of gunshots fired.
- Number of gunshots that successfully hit the AI agents.
- Number of times the participant got shot.
- Number of teleports issued and the distance teleported.

We did not record the number of enemies killed by each user. This was partly because we were already collecting data regarding successful gunshots. But more importantly, the kill count would not necessarily reflect the actual competency of the player. For example, one can imagine a scenario where an AI agent dealt most of the damage to an enemy, but the players fires the final shot and that would add the kill to their history, though the player only hit the enemy once. The following data were collected by sampling the system every 0.1 s:

- The participant’s virtual position in the VE.
- The HMD’s position and orientation.
- Thumbstick input (only relevant for continuous locomotion).
- The controllers’ position and orientation.

#### 3.7.2 Subjective Data.

Once the experiment finished, each user filled out a usability questionnaire. We collect basic demographic information including experience with using VR. We measure the usability of each locomotion method used using four sub-components: efficiency, learnability, likability, and accuracy [18]. Questions relating to the user’s locomotion mode were formulated as *“[Method] allowed me to navigate efficiently,” “[Method] allowed me to navigate with a high accuracy,”* and so on. Users had to answer the questions on a 5-point scale with a 1 being “strongly disagree” and a 5 being “strongly agree.” To assess the participants’ perception of the locomotion methods used by the AI, we added three more questions per method that had 5 levels: 1. *“Rate how difficult it was to hit enemies using [method]”* with responses ranging from “very hard” to “very easy”; 2. *“Compared to non*-*VR 3D games, the behavior of enemies that used [method] was:”* with responses ranging from “very unpredictable” to “very predictable”; and 3. *“I liked enemies navigating using [method]”* with responses ranging from “strongly disagree” to “strongly agree.”

## 4 RESULTS

We analyzed the following metrics for each locomotion technique:

1. $\text{Shooting}\;\text{accuracy}=\frac{\text{Number}\;\text{of}\;\text{shots}\;\text{that}\;\text{hit}\;\text{enemy}}{\text{Number}\;\text{of}\;\text{shots}\;\text{fired}}$
2. Hits taken, measured as the total number of enemy shots that hit the user.
3. $\text{Normalized}\;\text{stationary}\;\text{time}=\frac{\text{Total}\;\text{duration}\;\text{user}\;\text{was}\;\text{stationary}\;\text{in}\;\text{combat}}{\text{Total}\;\text{duration}\;\text{of}\;\text{trial}}$
4. Total change in head rotation around the global up direction.
5. Total physical movement in the tracked area.
6. Total virtual movement.
7. Total physical movement in the tracked area while the user was engaged in combat.
8. Total virtual movement during combat.
9. Physical movement standard distance [10]; a measure of physical movement spread. The standard distance is a measure of how spread out a point cloud is from the centroid.
10. Virtual movement standard distance: a measure of virtual movement spread.
11. Head gaze–locomotion direction angle; a measure of to what extent users feel the need to look in the direction they are going.
12. Mean teleportation distance.

**Fig. 3.**
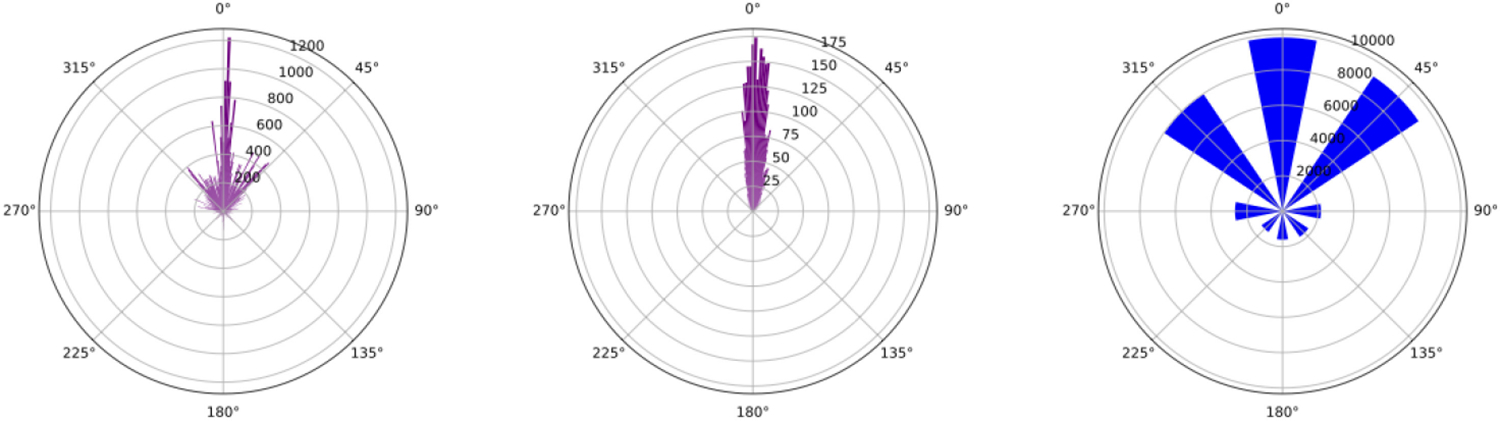
Radial histograms of the angle between head gaze and locomotion direction for (left) continuous locomotion and (middle) teleportation across all users. Angle for continuous was sampled whenever there was joystick movement. Angle for teleportation was sampled whenever a teleport was performed. (Right) Histogram shows the joystick input, divided into eight directions.

Table 2 lists the results. A repeated measures *t*-test was used to analyze for significant differences between the 12 aforementioned metrics. There was no significant difference between locomotion techniques for shooting accuracy (*T*_20_ = .49, *p* = 0.629. We found statistically significant differences between locomotion techniques for hits taken (*T*_20_ = 4.73, *p* < 0.001), normalized stationary time (*T*_20_ = 7.44, *p* < 0.001), total change in head rotation (*T*_20_ = 2.64, *p* = 0.016), total physical movement (*T*_20_ = 4.61, *p* =< 0.001), total virtual movement (*T*_20_ = 4.70, *p* =< 0.001), total physical movement in combat (*T*_20_ = 5.02, *p* =< 0.001), total virtual movement in combat (*T*_20_ = 4.19, *p* =< 0.001), physical movement standard distance (*T*_20_ = 2.40, *p* = 0.027), and virtual movement standard distance (*T*_20_ = 2.21, *p* = 0.019). A Wilcoxon signed-rank test for the angle between head gaze direction and locomotion direction found a significant difference (*Z*_20_ = 35.314, *p* < 0.001) between locomotion modes. Figure 3 shows radial histograms of these values. Finally, across all participants, the mean teleportation distance was calculated to be 5.85 m (*σ* = 3.39 m).

### 4.1 Subjective Results

**Fig. 4.**
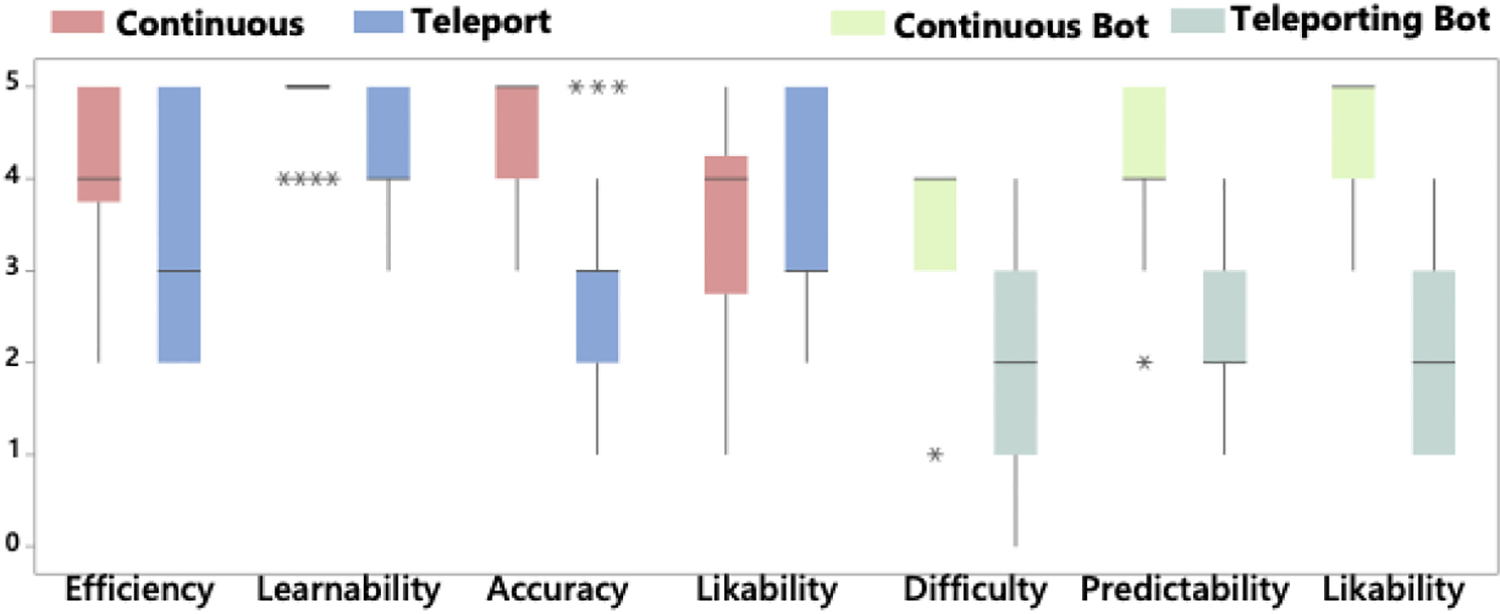
Columns show Likert scores (scale 1–5) as the median, the interquartile range, range, and outliers (denoted with *) for locomotion modes.

We first asked users to rate the locomotion methods they used and then to rate the agent’s AI navigation behavior using their locomotion method on a 5-point Likert scale. Results are listed in Figure 4. A Wilcoxon signed-rank test found no significant difference between efficiency (*Z*_20_ = −1.015, *p* = 0.31) and likability (*Z*_20_ = −.265, *p* = 0.791). Statistically significant differences were found between the median ranks for both learnability (*Z*_20_ = −2.546, *p* = 0.011) and accuracy (*Z*_20_ = −3.5, *p* < 0.001). For the perception of the AI locomotion, significant differences in median ranks were found for difficulty (*Z*_20_ = −3.479, *p* < 0.001), predictability (*Z*_20_ = −3.875, *p* < 0.001), and likability (*Z*_20_ = −4.045, *p* < 0.001).

## 5 DISCUSSION AND LIMITATIONS

The results of our study revealed some interesting insights and surprising findings.

### 5.1 Navigation Behavior

Based on our results, we accept our first hypothesis, **H**_**1**_, which stated that there were differences in navigation behavior. Looking into the movement data, teleportation users traveled about 46% farther virtually and 18.6% farther physically than when using continuous. At the same time, total head rotation was significantly higher (18.2%) for teleportation. While rotating one’s head, usually there is some positional displacement as well, which could have led to some higher physical movement. When looking at the mean angle between the users’ head gaze and the direction of travel, teleportation has significantly lower values (i.e., 23.9° versus 70.5° for continuous). This difference is also clearly visible in Figure 3. This implies that users tended to look in the direction where they wanted to go with teleportation but not with continuous locomotion, at least to the same extent. Teleportation involves a target selection task, so users are obviously looking at the reticle that defines their direction of travel. At the same time, we believe due to the discrete nature of teleportation, it requires users after each teleport to rotate to make small corrections to their heading, as they iteratively travel to a destination. These rotations over time accumulate to be larger than the head rotations required to navigate to the same destination using continuous locomotion. Though slower than teleportation, continuous locomotion offers a distinct advantage in letting users decouple their head orientation from the direction of travel. An analysis of joystick input for continuous locomotion (see Figure 3) reveals that forward (23%), forward+left (21.4%), and forward+right (18.6%) constitutes 63% of all input provided. Pure lateral strafing constituted 11% of input providing further evidence of how important this type of input is for FPS games. This limitation of teleportation has implications for game design; it requires players to look in the direction of travel, which means they have to take their eyes off important things, like an enemy or monster, which could be detrimental to performance. Though strafing while using regular teleport is not physically impossible, our results clearly suggest that users generally do not exhibit such behavior, likely because they feel the need to look in the direction they are pointing the teleportation arch at.

**Fig. 5.**
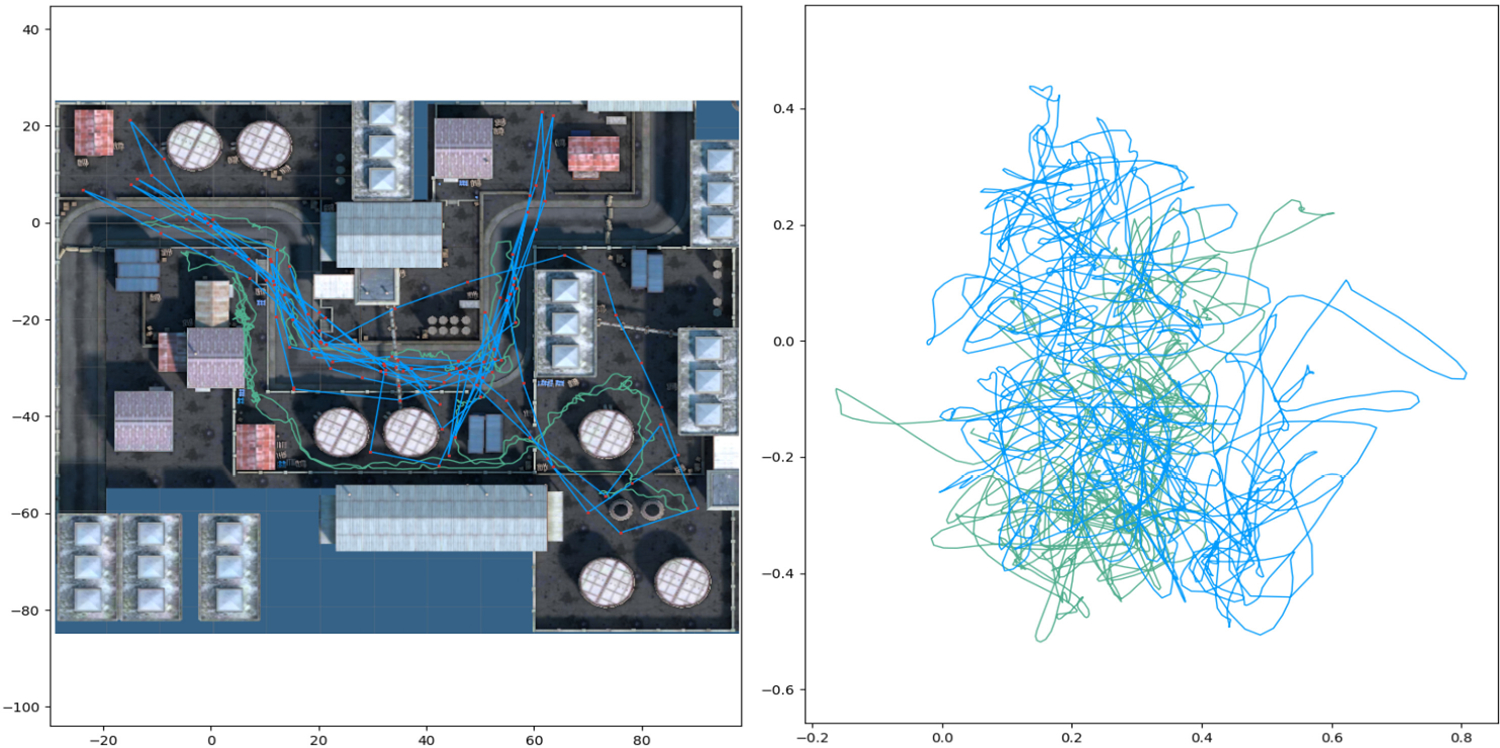
Example path trace (participant #4) in the VE (left) and in the tracked room space (right). Units are in meters. Blue traces are for teleport while Green is for continuous.

Participants traveled much larger virtual distances using teleportation. The higher virtual movement standard distance indicates participants traveled further toward the edges of the VE. This difference is also shown in Figure 5: left, which shows example path traces for participant #4. We designed the VE to be complex with varying elevations, walls, and fences so that it is similar to VEs found in traditional FPS games. Because of this layout, reaching even seemingly close-by locations is much harder for continuous locomotion and takes more time than teleportation, which lets participants jump over or through obstacles. Our findings contradict the results by Griffin et al. [24] who found teleportation users to travel less far. We believe this difference is explained by the fact that for research purposes they paired the locomotion speeds of both methods (i.e., traveling using teleportation and continuous locomotion took the same amount of time). Their VE was also a large open arena with no obstacles, which may not be representative of VEs found in FPS games. In our study, participants explored the VE more using teleportation, which seems to contradict the conventional notion that teleportation limits exploration because users can teleport to any location.

The ratio between the physical and the virtual movement for continuous (3.7%) and teleportation (2.9%) is low. Given that some physical movement is caused by head rotations, the actual ratios are likely even lower, which suggests that players mostly abandon physical walking while using both locomotion methods. This might indicate that artificial locomotion techniques tend to discourage physical walking and this is also in congruence with earlier findings by Prithul et al. [35]. Looking into the tracking space utilization, we found that all participants stayed within a 2.03 × 1.75 m tracked region throughout the experiment. A 2.5 × 2.5m tracking space seems sufficiently large to enable playing FPS games using teleportation and/or continuous locomotion.

### 5.2 Performance

We also accept our second hypothesis: **H**_**2**_ i.e., that differences in performance exist. For performance, we consider shooting accuracy and the number of times the participant got shot. We did not find a difference in accuracy that could be attributed to the fact that our AI agent was designed in such a way that they behaved identically for both locomotion methods used in our study. However, there was a significant difference between methods for the number of times participants got shot. On average, participants got hit nearly twice as much using teleportation. Looking at the normalized stationary times, we see that when participants were engaged in combat, they stayed stationary significantly longer with teleportation. Looking at the normalized stationary time for the two locomotion modes reveals that during combat, participants remained stationary twice as long with teleportation than when using continuous locomotion. A lack of strafinglike behavior while using teleportation could explain this difference. Participants travel faster and further using teleportation that could have exposed them to more enemies but we do not think this explains the difference in getting hit more.

Our results contradict results by Freitag et al. [19] who found that participants using continuous locomotion were hit more frequently. One key difference comparing our results to theirs is that we are using a hitscan-based, multi-agent, FPS game. Conversely, theirs was a projectile-based snowball fighting game with only two human players, each restricted to their own area. Keeping track of one human opponent and one snowball at a time may have been much simpler than avoiding multiple instant-hitting bullets from multiple AI agents. In addition, continuous locomotion allows for accurately predicting a player’s trajectory that helps with aiming projectiles, which could explain a higher hit rate for continuous locomotion. Our findings are consistent with Griffin et al. [24] who also used an AI-based multi-agent FPS game. This suggests that the discrepancy in results is caused by the fundamental difference between the two game types.

### 5.3 Perception

Finally, we also accept **H**_**3**_, which stated that there were differences in perception. According to the subjective results, there was no indication that participants preferred one locomotion technique over the other or found one to be more efficient than the other. However, they did express that continuous locomotion was considerably easier to learn and allowed for travel with much greater accuracy. When it comes to the perception of locomotion techniques used by other users (AI agents in our study), users largely preferred continuous locomotion. Contrary to our empirical results, participants believed that they were less accurate when shooting AI agents who were using teleportation. Furthermore, participants perceived the agents using teleportation as more unpredictable compared to those using continuous locomotion. This perception was reflected in their lower likability scores assigned to the teleporting AI agents. The fact that avatars were discontinuously represented may have contributed to a feeling of unpredictability.

### 5.4 Limitations

Our study specifically focused on understanding differences in performance, navigation behavior, and perception of both locomotion techniques. We did not evaluate differences in presence (though perception is closely related), VR sickness, or other factors like spatial disorientation. Differences in presence and VR sickness are well known and studied (see Reference [34]) and spatial disorientation when using teleportation can be mitigated by adding a small amount of optical flow to the viewpoint transition [5]. Our study did not let participants die in the game so as to not penalize less experienced players. Though participants were not even informed they could die, it is possible that their behavior would be different if death was used as a reinforcement mechanism. However, it should also be mentioned that our results show that users did try to avoid getting shot as demonstrated by the much lower number of hits taken when using continuous locomotion. Future work may explore using a negative points systems as an alternative to dying.

To ensure a controlled and replicable user study, we opted to utilize AI agents instead of human participants. While this decision allowed us to maintain consistency and repeatability, it is important to note that our findings still provide valuable insights into the effects of various locomotion methods on multiplayer experiences. Despite our diligent efforts to recruit a diverse pool of participants, including a specific focus on including women, we unfortunately encountered a significant gender imbalance in our final participant distribution. We acknowledge and are mindful of this disparity in our study, which seems common to many VR studies [32].

One of the significant challenges we encountered during the design of our AI agents was the lack of knowledge regarding how FPS players navigate. In fact, gaining a deeper understanding of navigation behavior was one of the primary objectives of our study. By addressing this knowledge gap, our research contributes to the broader understanding of player behavior in FPS games and sheds light on the impact of different locomotion techniques. Our results show that participants who utilize teleportation tend to remain stationary, even though they have the option to physically strafe. Our AI agents displayed strafing behavior for both locomotion methods to keep conditions the same in the absence of any prior study on which to base our design on. To more closely align with observed human behavior, it would be necessary to eliminate strafing behavior from AI agents that use teleportation. This would increase their vulnerability to being hit, leading to a discrepancy in shooting accuracy between the two locomotion methods. In that case, the result regarding shooting accuracy likely will show that AI bots also get hit more often when using teleport, similar to human users. Additionally, we lacked information on the typical distance that players would teleport. As a result, we set the median teleportation distance for the AI agents to 7 m. Interestingly, this value was not too far from the actual average distance that participants used, which was 5.85 m. It is worth noting that participants had the ability to teleport up to 18 m. The insights garnered from our study will play a pivotal role in the development of AI agents capable of demonstrating more realistic navigation behavior. By understanding the impact of different locomotion methods on player experiences, we can inform the design and training of AI agents to enhance their ability to navigate virtual environments in a manner that closely resembles human behavior. We conducted our study in a particular VE that we considered representative of FPS games. However, we acknowledge that its unique design may have influenced navigation behavior in a biased manner. In future research, we intend to investigate different VEs to understand if level design affects navigation behavior. In addition to our current study on FPS games, future research endeavors will expand their focus to encompass other types of games to gain a broader understanding of how different locomotion methods impact player experiences across various gaming contexts.

### 5.5 Gameplay Design Considerations

Whether to offer teleportation or continuous locomotion in any VR experience may involve various tradeoffs regarding presence and performance. Given that teleportation can enable access to a much larger group of people, it should be considered to be an accessibility feature and it is reasonable to assume that multiplayer VR games will offer both locomotion methods (which is already the case with popular VR games like Rec Room^8^). Balancing gameplay between both locomotion modes can be quite a challenge but game designers could consider the following issues based on the findings of our study:

- When avatars are discontinuously represented it can add to a feeling of unpredictability and make their paths hard to predict. Thus avatars should be represented continuously. Several VR apps (i.e., From other Suns^9^ as well as VR Chat^10^) offer mechanisms where a user can point at a location and your avatar will be shown to walk to this location and the viewpoint moved when the avatar arrives. Several other solutions have been proposed to allow for a continuous avatar representation [20, 23].
- Regular teleportation does not encourage strafing as we found out from our study. Thus, designers might consider modifying it somehow to allow or encourage strafinglike behavior. It may be challenging to facilitate but this could bridge the gap in performance regarding the number of hits taken between teleportation and continuous locomotion.
- The VE should be designed to mitigate any differences in locomotion methods, i.e., being able to teleport through a fence versus having to go around it using continuous locomotion creates a huge imbalance in gameplay and should be avoided. Teleportation could be modified such that a target destination can only be selected if a collision-free path to this destination exists. An example of such a teleportation mechanism can be found in the game spell fighter VR.^11^

## 6 CONCLUSION

Though teleportation is currently one of the most widely used locomotion methods in VR, it is unclear how its usage affects the performance and navigation behavior of users, particularly for FPS games, which is a popular game genre. To investigate this, we conducted a user study with 21 participants to compare teleportation versus continuous locomotion using an FPS shooter game in which other players were simulated using AI agents. The study found that while there was no significant difference in shooting performance between the two methods, users who used teleportation traveled longer distances but were mostly stationary during combat and were hit more frequently as a result. These differences have significant implications for balancing gameplay between the two locomotion methods, and we discuss potential strategies for achieving this balance.

## Figures and Tables

**Table 1.** Number of Participants with Experience Level Ranging from “No” (Users Had No Experience) to “Lots” (Users Had Lots of Experience)

	No				Lots
Experience with using VR	0	1	4	8	8
Experience with using teleportation in VR	1	5	3	6	5
Experience with multiplayer games	3	0	1	1	17
Experience with multiplayer VR games	5	2	5	2	4

**Table 2.** Performance and Behavioral Results

Locomotion	Continuous (SD)	Teleport (SD)	Sig diff
Shooting accuracy	0.368 (0.07)	0.363 (0.07)	No
Hits Taken	128.524 (70.94)	249.048 (116.04)	Yes
Normalized stationary time	0.111 (0.14)	0.226 (0.15)	Yes
Change in head rotation (degrees)	18,832 (5,291)	22,276 (5,599)	Yes
Physical movement (meters)	27.911 (6.84)	33.112 (6.88)	Yes
Virtual movement (meters)	754.447 (224.93)	1104.074 (442.32)	Yes
Physical movement in combat (meters)	12.122 (4.03)	19.11 (5.48)	Yes
Virtual movement in combat (meters)	544.838 (191.95)	832.032 (381.98)	Yes
Physical movement standard distance (meters)	0.176 (0.04)	0.205 (0.06)	Yes
Virtual movement standard distance (meters)	20.302 (8.30)	24.962 (7.10)	Yes
Gaze-locomotion angle (degrees)	70.473 (51.38)	23.965 (27.26)	Yes
Mean teleport distance (meters)	–	5.850 (3.39)	N/A

## Box

CCS Concepts: • **Human-centered computing** → **Virtual reality**;

## Footnotes

1
https://www.callofduty.com/blog/2021/04/Incredible-Warzone-and-Game-Sales-Milestones

2
https://www.digitaltrends.com/computing/vr-locomotion-movement-omni-hover-junkers/

3
https://www.uploadvr.com/half-life-alyx-2-million

4
https://assetstore.unity.com/packages/3d/environments/industrial/rpg-fps-game-assets-for-pc-mobile-industrial-set-v2-0-86679

5
https://www.reddit.com/r/Vive/comments/4fqq4a/vr_roomscale_room_size_survey_answers_analysis/

6
https://docs.unity3d.com/Packages/com.unity.xr.interaction.toolkit@2.2/manual/index.html

7
https://github.com/SixWays/UnityVrTunnelling

8
https://recroom.com

9
https://gunfiregames.com/games/fromothersuns

10
https://hello.vrchat.com/

11
https://store.steampowered.com/app/455440/Spell_Fighter_VR/
